# Social Support, Adjustment, and Psychological Distress of Help-Seeking Expatriates

**DOI:** 10.5334/pb.464

**Published:** 2018-10-05

**Authors:** Mojca Filipič Sterle, Tine Vervoort, Lesley L. Verhofstadt

**Affiliations:** 1 Department of Experimental-Clinical and Health Psychology, Ghent University, Ghent, BE; 2 Department of Marital and Family Therapy, Faculty of Theology, University of Ljubljana, Ljubljana, SI

**Keywords:** expatriates, cross-cultural adjustment, social support, socio-emotional support, instrumental support, help-seeking sample

## Abstract

The present study aimed to explore the interrelations between support processes, adjustment, and psychological distress within a sample of help-seeking expatriates. Specifically, we examined (1) the association between expatriates’ cross-cultural adjustment (i.e., work, interaction and general adjustment) and levels of psychological distress (i.e., depression, anxiety and stress), (2) the association between expatriates’ perceptions of socioemotional and instrumental support availability and their level of cross-cultural adjustment, and (3) the moderating role of expatriates’ socioemotional and instrumental support needs in the latter association. Findings showed that lower levels of expatriates’ work adjustment were associated with higher levels of psychological distress. Further, perceived availability of socioemotional support was positively linked to expatriates’ interaction and work adjustment. Finally, instrumental support needs moderated the relationship between instrumental support availability and general adjustment such that higher levels of instrumental support availability were associated with better general adjustment, but only for expatriates reporting high needs for instrumental support. Our study represents a novel contribution to the expatriate literature by shedding light on expatriates’ vulnerability for psychological distress and understanding the type of social support that is considered most beneficial for help-seeking expatriates. Suggestions are made for clinical interventions for expatriates in need of support.

## Introduction

There is a growing acknowledgement in the research literature that international work can be a challenging and stressful experience and that psychological support in the host country may alleviate distress for expatriates and facilitate their initial transition (Lazarova, McNulty & Semeniuk, 2015; Rosenbusch & Cseh, 2012). However, there is little or no research available that investigates expatriate adjustment and social support when things get really difficult for them and they are in need for help. Examination of the type of social support for expatriates who are experiencing some psychological distress is particularly important because it can provide the evidence of how expatriates can be best assisted in their host country. The current study aimed to contribute to the current research literature by studying cross-cultural adjustment, support needs, and support availability, in a sample of expatriates seeking psychotherapy and experiencing heightened levels of psychological distress (e.g., depression, anxiety and stress). More specifically, we examined: (1) the association between expatriates’ cross-cultural adjustment (i.e., work, interaction and general adjustment) and levels of psychological distress (i.e., depression, anxiety and stress); (2) the association between expatriates’ perceptions of socioemotional and instrumental support availability and their level of cross-cultural adjustment; and (3) the moderating role of expatriates’ socioemotional and instrumental support needs in the latter association. We drew upon theoretical perspectives on expatriate adjustment (Bhaskar-Shrinivas, Harrison, Shaffer, & Luk, 2005; Black, Mendenhall & Oddou, 1991; Black & Stephens, 1989; Filipič Sterle, Fontaine, De Mol, & Verhofstadt, 2018a; Haslberger, Brewster & Hippler, 2013; Hippler, Brewster & Haslberger, 2015; Ward, Bochner & Furnham, 2001), the stress and coping literature (Lazarus & Folkman, 1984; Patterson, 1988; Seyle, 1978), social support theories with attention to social support availability and social support needs (Cutrona & Russell, 1990; Melrose, Brown & Wood, 2015; McGinley, 2008; Ong & Ward, 2005; Podsiadlowski, Vauclair, Spiess & Stroppa, 2013; Rafaeli & Gleason, 2009; Selmer, 1999), as well as literature on clinical interventions for the globally mobile (Bushong, 2013; Filipič Sterle, Verhofstadt, Bell & De Mol, 2018b).

### Expatriate Adjustment and Psychological Distress

An *expatriate* is generally defined as a person who lives or works outside his or her own home country on a non-permanent basis (Andreason, 2003; Ward et al., 2001). Apart from most studied company-based expatriates (Gonzalez-Loureiro, Kiessling & Dabic, 2015), expatriates are also highly skilled professionals or knowledge workers who actively and voluntarily pursue employment opportunities abroad and tend to maintain a degree of flexibility and freedom in their career choice, work location and family-work balance (Adams & van de Vijver, 2015; McNulty, 2013, 2014). Recent research has offered a multidimensional approach to define different types and dimensions of international work experience (Baruch, Dickmann, Altman & Bournois, 2013), including alternative or non-traditional expatriates pursuing global careers (see e.g., McNulty, 2015). A common characteristic of these expatriates is that rather than being linked to local culture, they take on a global mindset and become part of an international, cross-cultural social environment (Mao & Shen, 2015).

When expatriates move to a foreign country, they start to learn about the host culture and make efforts to find successful ways of functioning in a host country. This process is called cross-cultural adjustment and according to the conceptualization proposed by Black and Stephens (1989) it consists of three facets of adjustment. A degree of familiarity with functioning in the host culture, such as health care, housing conditions, costs of living, etc., describes expatriates’ level of overall adjustment to living in the new culture, called general adjustment (Black & Stephens, 1989). Work adjustment relates to expatriates’ adjustment to a new work situation with co-workers and new work tasks and work roles (Black & Stephens, 1989) which expatriates may experience as a result of new career opportunities (Dickmann, Doherty, Mills & Brewster, 2008). Interaction adjustment is a process associated with social interactions and communication with other people in the host culture and with building new social networks (Black & Stephens, 1989). Based on this Black and Stephen’s conceptualization of expatriate adjustment, expatriates are considered adjusted to a foreign culture when they experience familiarity and psychological comfort or absence of stress on the three dimensions of expatriate experience (Black & Stephens, 1989). Differentiation between the three dimensions of adjustment is important as it allows the examination of factors that predict the adjustment processes on each of the three dimensions.

Within the expatriate adjustment literature, it has been recognized that the process of adjustment in the host country has a significant impact on the success of an international work experience (Black et al., 1991). The static perspective has long been the main stream in the research agendas on expatriate adjustment, thereby neglecting the time dimension and the variability of adjustment over time. Some recent research, however, documents the importance of considering the aspect of “time” when looking at the process of adjustment (see e.g., Firth, Chen, Kirkman & Kim, 2014), and to consider the specifics of adjustment processes on the three dimensions described above (Hippler et al., 2015).

Adjustment issues are indeed central in expatriates’ lives as international work experience involves multiple changes and new experiences and challenges (Truman, Sharar & Pompe, 2012). In accordance with the adjustment theoretical models and the stress literature, an expatriate experience constitutes a major life event involving a variety of specific stressors (Lazarus & Folkman, 1984; Patterson, 1988), such as starting a new job, moving abroad, a partner giving up a job, children attending a new school, occupying a new residence, change of family routines and financial status, cultural differences (Haslberger & Brewster, 2008; Patterson, 1988), long periods of separation from loved ones (Bahn, 2015), and disruption of an expatriate’s social support system (Fontaine, 1986). The attempt to reduce initial stress triggers coping responses which result in better adjustment and increased subjective well-being (Black & Stevens, 1989; Haslberger et al., 2013).

Failure of managing change and stress may compromise expatriate adjustment, thus resulting in deteriorating mental health and well-being. For example, some empirical evidence points at the deleterious psychological correlates of expatriation, including heightened psychological distress (Anderzén & Arnetz, 1999; Forster, 1997; Foyle, Beer & Watson, 1998; Silbiger & Pines, 2014; Truman et al. 2012), decreased mental well-being, a worse subjective work environment (Anderzén & Arnetz, 1999), more alcohol and substance abuse (Anderzén & Arnetz, 1997; Truman et al., 2012), externalizing problems, such as attention deficit, hyperactivity, impulse control (Truman et al., 2012), and increased depression (Magdol, 2002; Truman et al., 2012). Acknowledging adjustment as a process that happens on different dimensions – general life, interaction with locals, and work (Black & Stevens, 1989), and adjustment being specific on each dimension (Haslberger et al., 2013), the current study aimed to contribute to the understanding of the association between the three dimensions of adjustment and psychological distress. Our first hypothesis was formed as follows:

Hypothesis 1: Lower levels of (general, interaction and work) adjustment will be associated with higher levels of psychological distress.

### Social Support and Coping

Expatriates may rely on intrapersonal and interpersonal resources to cope with stressors and challenges and to overcome culture shock, as stated in the stress literature (Patterson, 1988). Emerging evidence points at the key role of interpersonal variables, more specifically *social support* (Adelman, 1988; Fontaine, 1986). Empirical findings show that increased social support contributes to better adjustment of expatriates (Adelman, 1988; Attaca & Berry, 2002; Aycan, 1997; Black & Gregersen, 1991; Caligiuri & Lazarova, 2002; Fontaine, 1986). The research literature on social support in the expatriate context identifies socioemotional support and instrumental support (Caligiuri & Lazarova, 2002). Socioemotional support can alleviate negative feelings and facilitate expatriate adjustment through emotional support networks in the host country (Farh, Bartol, Shapiro & Shin, 2010). It provides expatriates with feelings of belonging and psychological security and thus addresses their needs to affiliate, for contact, sharing, companionship and friendship (Caligiuri & Lazarova, 2002). Instrumental support can also ease stressful situations by fulfilling specific needs (Caligiuri & Lazarova, 2002). Examples of such needs are help with the children, help with works in a new home, etc. (Ong & Ward, 2005). Moreover, instrumental support can increase feelings of connectedness and acceptance by the local community, therefore increase the interaction adjustment (Shumaker & Brownell, 1984). In attempts to identify the role of a particular type of social support for expatriates’ successful adjustment, the research has shown diversified empirical evidence. In particular, some studies have pointed at the key role of instrumental support (McGinley, 2008; Ong & Ward, 2005; Selmer, 1999), whereas other studies suggested socioemotional support being more important for successful adjustment (Farh et al., 2010; Podsiadlowski et al., 2013). However, these studies used samples of expatriates who were not seeking psychological help. Our study aimed to fill this research gap by exploring the impact of social support on the three dimensions of adjustment within a sample of expatriates seeking psychotherapy. As such, it should allow a reexamination of the positive impact of social support on adjustment, as described in research using non-clinical samples of expatriates.

*Hypothesis 2: Higher levels of perceived social support availability* (*socioemotional and instrumental support*) *will be associated with better general, interaction and work adjustment.*

Further, emerging evidence in the general support literature documents that support provision is only helpful when it matches the recipient’s subjective need for a specific level/type of support (Cutrona & Russell, 1990; Melrose et al., 2015; Podsiadlowski et al., 2013; Rafaeli & Gleason, 2009), and that social support interventions may only be helpful if the individuals are in need of that particular type of support but not when these needs are already being met or are not at stake (Melrose et al., 2015). Matching and mismatching support have been found to differentially impact outcomes. For instance, when a particular type of support matches one’s support needs it may be helpful for recipient. Mismatched support (support that is not needed or provided too early), on the other hand, may have an undermining effect on the recipients’ sense of autonomy, competence and self-esteem, or may signal that they cannot cope with the situation independently (Rafaeli & Gleason, 2009). Further, mismatched support can also aggravate feelings of indebtedness and violate the sense of reciprocity (i.e., recipients feel indebted to other people offering the support) and may also imply increased and potentially unwanted attention to strains and hardships of their life (Rafaeli & Gleason, 2009). Based on the above reasoning, our third hypothesis was formed as follows:

Hypothesis 3: Higher levels of perceived instrumental and socioemotional support availability will contribute to all three dimensions of adjustment, particularly for expatriates reporting high respective support needs.

## Method

### Participants

Participants were solicited by the help of mental health professionals (i.e., psychologists and psychotherapists) working in Brussels (Belgium). A total of 31 psychotherapists who offer psychotherapy treatment to expatriates were contacted either by person, e-mail or telephone and were requested to inform their clients about the present research. Of these, 16 psychotherapists agreed to talk to their expatriate clients and invite them to take part in the current study. The main reasons for psychotherapists to refuse participation was lack of time, clients’ non-compliance with their psychotherapy approach, and in some cases no particular reason was provided. Inclusion criteria were a) being an expatriate living in Brussels or the surrounding areas, b) seeing a psychotherapist at the time of the study, c) having reached adult age (i.e., 18+), and d) sufficient fluency in English in order to fill out the questionnaires. Of 353 expatriates eligible to participate, 214 agreed to take part. Missing values analyses (i.e., more than 25% of the items of a given questionnaire not answered) indicated that complete data were available for 97 participants. No information was available on reasons for not participating.

Participants in our study shared the characteristic that they were actual clients in psychotherapy, thus representing a sample of expatriates seeking professional help. The majority of participating expatriates were female (*N* = 78; 80%). The mean age of the participants was 40.1 years (*SD* = 9.1, *range* = 19 years to 70 years) and the average duration of their stay in Belgium was 8.7 years (*SD* = 7.4, *range* = 0 to 42 years). Forty-four percent of the expatriates were married (*n* = 43), 31% were single (*n* = 30), 12% were cohabiting with their partner (*n* = 12); 6% were divorced (*n* = 6); for 7% (*n* = 6) of the participants, no data on their relationship status was available. The majority of expatriates – 69% – had a university degree (*n* = 67), 14% finished college (*n* = 13), followed by 9% who had an academic degree (*n* = 9), 6% finished high school (*n* = 6), and 1% finished primary school (*n* = 1). No data was available for 1% of the participants (*n* = 1). Seventy-one percent of the participants had an open-ended work contract (*n* = 69). About half (52%) of the sample was working for the European institutions (*n* = 50). The majority of respondents – 69% initially moved to Belgium because of a work assignment (*n* = 67), whereas 28% followed their spouses as “trailing partners” (*n* = 27). Expatriate participants were coming from 26 countries of origin and were categorized in three regional groups: Western Europe (e.g., UK, France, Germany, Italy, etc.) (57%, *n* = 56), Eastern Europe (e.g., Slovenia, Hungary, Estonia, Romania, etc.) (32%, *n* = 31), and non-European countries (e.g., USA, Australia, Brazil, Turkey, etc.) (10%, *n* = 9). More specifically, participants came from Slovenia (13%; *n* = 12), UK (11%; *n* = 10), Ireland (9%; *n* = 7), Germany (10%; *n* = 9), Sweden and Poland (5%; *n* = 5 each), Romania, Hungary and Italy (4%; *n* = 4 each), Finland, Estonia, France, and USA (3%; *n* = 3 each), Australia, Austria, The Netherlands, Portugal, and Turkey (2%; *n* = 2 each). The remainder of the participants (13%; *n* = 15) came from other, mostly European countries. Sixty-six percent of the respondents of our clinical sample had not been given a DSM diagnosis by their treating doctors (*n* = 61). Thirty percent were diagnosed by a doctor or a psychiatrist (*n* = 28), 12% as suffering from depression (*n* = 11).

### Measure

**Cross-cultural adjustment.** Black and Steven’s cross-cultural adjustment scale was used to assess participants’ adjustment to life in Belgium (Black & Stephens, 1989). This widely used 14-item scale has demonstrated high reliability across multiple samples (Black & Gregersen, 1991; Black & Stevens, 1989). Respondents were asked to indicate how unadjusted or adjusted they currently felt to their life in Belgium by means of a 7-point Likert rating scale (1 = *completely unadjusted* to 7 = *completely adjusted*). The *General adjustment* subscale measures perceived adjustment in everyday life (seven items; e.g., “living conditions in general”). The *Interaction adjustment* subscale measures the expatriate’s interaction with host country nationals (four items; e.g., “interacting with Belgians on a day-to-day basis”). The *Work adjustment* subscale measures expatriate’s adjustment to new job tasks, environment, and roles (three items; e.g., “performance standards and expectations”). Subscale scores were computed by averaging item scores, with higher scores indicating higher levels of adjustment. In our study Cronbach’s *alphas* were .89 for general adjustment, .93 for interaction adjustment, and .93 for work adjustment.

**Psychological distress (depression, anxiety and stress).** Participants’ level of psychological distress was assessed by the DASS-21 (Lovibond & Lovibond, 1995) which measures the severity of the core symptoms of depression, anxiety and stress. The *Depression* subscale assesses the symptoms of dysphoria, hopelessness, devaluation of life, self-deprecation, lack of interest or involvement, anhedonia and inertia (seven items, e.g., “I felt that I had nothing to look forward to”). The *Anxiety* subscale assesses autonomic arousal, skeletal muscle effects, situational anxiety, and subjective experience of anxious affect (seven items, e.g., “I felt scared without any good reason”). The *Stress* subscale is sensitive to levels of chronic non-specific arousal and assesses difficulty relaxing, nervous arousal, and being easily upset/agitated, irritable/over-reactive and impatient (seven items, e.g., “I tend to over-react to situations”). In the current study, participants were required to indicate the presence of a symptom over the last two weeks by means of a 4-point severity/frequency Likert scale (1 = *Did not apply to me at all* to 4 = *Applied to me very much or most of the time*). Subscale scores were computed by averaging the scores for the relevant items. As the three subscales of the DASS-21 were highly correlated in the current study (i.e., correlations ranging from *r* = .67 to *r* = .70; *p* < .01), we used the total DASS-21 score. The total DASS-21 score has been previously documented as an index for general psychological distress (Henry & Crawford, 2005). The Cronbach’s *alphas* were the following: depression subscale α = .92, anxiety subscale α = .73, stress subscale α = .84, overall DASS-21 scale α = .93.

**Perceived availability of support.** The Index of Sojourner Social Support was used to measure expatriates’ perception of the availability of supportive behaviours that serve particular types of functions (i.e., expatriate’s perception of people who could provide specific helpful behaviours for them in the new country) (Ong & Ward, 2005). This 18-item scale consists of two subscales: *Socioemotional support* defined as provision of feelings of belonging and psychological security that buffer against negative feelings (e.g., friendship, companionship) (nine items; e.g., “Comfort you whenever you feel homesick”) and *Instrumental support* defined as the creation of a supporting environment with provision of necessary resources that can ease stressful situations (e.g., help with children, help with works in the new home, etc.), (nine items; e.g., “Provide necessary information to help you orient you to your new surroundings”). Respondents were asked to indicate whether they knew people who would provide each of the specific helping behaviors for them in the host county. Items were rated using 5-point Likert scales ranging from 1 = *No one would do this* to 5 = *Many would do this*. Subscale scores were computed by summing scores for all items included in a specific subscale, with higher scores indicating higher levels of perceived availability of social support. The alpha reliabilities for the perceived availability of sojourner support were high (α = .96 for socioemotional support, and α = .95 for instrumental support).

**Support Needs.** In order to measure participants’ need for support, we used two subscales of the Brief COPE. This 28-item questionnaire is a widely used instrument to assess 14 different coping strategies that individuals tend to use when trying to face stress or dealing with problems (Carver, 1997). For the current study, only the *Seeking emotional support* and the *Seeking instrumental support* subscales were used. The *Seeking emotional support* subscale refers to an individual’s tendency to seek emotional support from others (two items; e.g., “getting comfort and understanding from someone”). The *Seeking instrumental support* refers to an individual’s tendency to seek instrumental support from others (two items, e.g., “getting help and advice from other people about what to do”). Respondents indicated the ways they had been dealing with problems and stress since they have lived in Belgium by means of a 4-point Likert scale (1 = *I haven’t been doing this at all* to 4 = *I have been doing this a lot*). Subscale scores were computed by summing scores for all items included the respective subscale, with higher scores indicating more support seeking (i.e., attempts to get their support needs met). In our study the Cronbach’s *alpha’s* were .85 for the *Seeking emotional support* subscale and .85 for the *Seeking instrumental support* subscale.

### Procedure

Eligible clients who expressed interest to participate in this study were offered an information letter and a confidential internet survey login code by their treating psychotherapist. Completion of the online survey took about 30–45 minutes. The survey was administered in English. The study was approved by the ethical committee of the Faculty of Psychology and Educational Sciences of Ghent University, Belgium, and The National Medical Ethics Committee of the Republic of Slovenia.

### Statistical Analyses

A hierarchical regression analysis (using SPSS 23.0) was conducted to examine the contribution of participants’ general, interaction and work adjustment in explaining psychological distress. Participants gender (men coded as 0, women coded as 1) and age were entered in step 1 to control for the effects of these socio-demographic variables upon psychological distress. In the second step, the number of months expatriates were living in Belgium was entered. In the third step, participants’ general, interaction and work adjustment were entered.

A series of 6 hierarchical regression analyses was conducted to examine the contribution of participants’ level of socioemotional/instrumental support availability in explaining adjustment and the moderating role of emotional/instrumental support needs. As with the previous analysis, participants gender (men coded as 0, women coded as 1) and age were entered in step 1; the number of months expatriates were living in Belgium was entered in step 2. To test for support needs as a moderator, it is necessary to enter the cross-product terms of support needs and availability in a separate block (step 4) in a hierarchical regression analysis, following the entry of support needs and availability as first-order terms (step 3). To reduce the effects of multicollinearity, continuous variables were centered (Holmbeck, 2002). Analyses were run separately for socioemotional and instrumental support availability and respective support needs in explaining either general, interaction or work adjustment. In case of significant interaction effects of support availability with support needs, additional moderation analyses were performed to interpret the interaction effect – i.e., whether the association between the predictor variable (socioemotional/instrumental support availability) and outcome variable (general/interaction/work adjustment) was significant at high or low (or both) levels of the moderator variable (emotional/instrumental support needs). Moderation analyses followed the procedure outlined by Holmbeck (1997, 2002). This procedure does not categorize participants into two groups but allows, by manipulating the 0 point of the moderator, to examine conditional effects of the continuous moderator variable upon the outcome. To this end, two steps were performed. First, two new conditional continuous moderator variables were computed by (1) subtracting 1 *SD* from the centred moderator variable (to compute high levels of socioemotional/instrumental support needs) and (2) adding 1 *SD* to the centred moderator variable (to compute low levels of socioemotional/instrumental support needs). Second, two additional regression analyses were performed – incorporating each of these new conditional continuous moderator variables – to test the significance for high and low values of the conditional moderator variable. Variance-inflation factors were acceptable (all VIF ≤ 2.8), suggesting that there was no problem of multicollinearity.

## Results

### Descriptive Statistics

Mean scores, standard deviations, and correlation coefficients for all measures are presented in Table 1. The average duration of stay was 8.7 years (*SD* = 7.4 years). 25% of the sample had a stay equal to or shorter than 3.4 years, 25% higher than 3.4 years and equal or shorter than 6.4 years, 25% higher than 6.4. years and equal or shorter than 10.9 years, and 25% equal to or shorter than 42.9 years.

**Table 1:** Means (M), standard deviations (SD), and Pearson correlations of expatriate general, interaction and work adjustment, psychological distress, socioemotional and instrumental sojourner support availability and emotional and instrumental support needs (*N* = 97).

	Range	M	SD	1		2		3		4		5		6		7		8

1. General adjustment	4.43	5.95	0.89															
2. Interaction adjustment	6	4.62	1.65	.45	**													
3. Work adjustment	6	5.67	1.41	.31	**	.24	*											
4. Psychological distress	102	35.63	23.09	–.30	**	–.19		–.53	**									
5. Socioemotional sojourner support availability	41	24.56	8.90	.20		.34	**	.21		–.24	*							
6. Instrumental sojourner support availability	36	25.41	8.28	.18		.28	*	.17		–.16		.69	**					
7. Emotional support needs	6	5.34	1.81	–.01		.25	*	–.03		–.02		.41	**	.44	**			
8. Instrumental support needs	6	5.65	1.66	–.04		.14		–.17		.04		.31	**	.43	**	.70	**	
9. Duration of stay	0–42 years	8.7 years	7.4 years	.31	**	.39	**	.20		–.28	**	.08		.03		–.04		–.04

* *p* < .05; ** *p* < .01.

According to the DASS-21 severity ratings (Lovibond & Lovibond, 1995), the current sample reported severe levels of psychological distress (i.e., depression, anxiety and stress) (*M* = 35.6, *SD* = 23.1 for overall DASS-21 score), as compared to the cut-off value of 32–38 described as indicating a severe degree of psychological distress.

### Correlations

Pearson correlation analysis revealed that expatriates’ general and work adjustment were significantly negatively correlated with psychological distress (*r* = –.30; *p* < .01 and *r* = –.53; *p* < .01, respectively) indicating that the expatriates who were not adjusted well to their everyday life in Belgium and their work environment reported experiencing higher levels of psychological distress. The association between interaction adjustment and psychological distress showed a low negative correlation but did not reach significance. Age and the duration of participants’ stay in Belgium were found to be highly correlated (*r* = .65; *p* < .0001). Some weak significant correlations were found between participants’ age and work adjustment, their psychological distress, and emotional support needs (all *r* ≤ .26; *p* < .05). The duration of stay was significantly negatively correlated with psychological distress (*r* = –.28; *p* < .01), indicating that expatriates who have lived in Belgium for a longer duration experience lower levels of psychological distress. Furthermore, a positive correlation was found between duration of expatriates’ stay in Belgium and general (*r* = .31; *p* < .01) and interaction adjustment (*r* = .39; *p* < .01). Participants’ levels of both emotional and instrumental support availability on the one hand and support needs on the other hand were significantly and positively correlated with each other (all *r* ≥ .31; *p* < .01). Further, the three cross-cultural adjustment subscales were all significantly correlated with each other (all *r* ≥ .24; *p* < .05); There was a strong positive correlation between our measures of socioemotional and instrumental support availability (*r* = .69; *p* < .01). By the same token, our measures of emotional and instrumental support needs were strongly and positively linked (*r* = .70; *p* < .01). There were no sex differences, except for emotional support needs (*t*(92) = 2.24; *p* < .05) which were found to be higher in women (*M* = 5.5) than in men (*M* = 4.5).

### Value of Expatriate Adjustment in Explaining Psychological Distress (Hypothesis 1)

A hierarchical regression analysis was conducted to examine the unique and potentially distinctive contribution of participants’ general, interaction and work adjustment in explaining psychological distress. A summary of this analysis is presented in Table 2. Participants gender (men coded as 0, women coded as 1) and age were entered in step 1 to control for the effects of these socio-demographic variables upon psychological distress. In the second step, the number of months expatriates were living in Belgium was entered. In the third step, participants’ general, interaction and work adjustment were entered simultaneously. The variance-inflation factors of this analysis were acceptable (range 1.21–2.78), suggesting that there was no problem of multicollinearity. Findings for the final model (step 3) indicated no significant effects for participants’ gender, age or time in Belgium (all |β|’s ≤ .09, ns). In line with our expectations, expatriate adjustment contributed significantly to psychological distress, yet, inspection of Beta Coefficients indicated that this was only the case for work adjustment; i.e., lower levels of work adjustment were associated with higher levels of psychological distress (*β* = –.47; *t* = –4.5; *p* < .0001). No significant contribution was observed for general and interaction adjustment (both β|’s ≤ .17, ns).

**Table 2:** Hierarchical regression analysis explaining psychological distress. Standardized betas from the last step in the analyses are displayed.

Criterion variable	Step	Predictor	*β*		R^2^Change		Adj R^2^	

Psychological distress	1	Age	–.09		.08	*	.05	*
		Gender	.07					
	2	Time in Belgium	–.06		.01		.05	
	3	General adjustment	–.17		.25	**	.28	**
		Interaction adjustment	.04					
		Work adjustment	–.47	**				

* *p* < .05; ** *p* < .0001.

### Value of Support Availability in Explaining Adjustment (Hypothesis 2) and the Moderating Role of Support Needs (Hypothesis 3)

A series of hierarchical regression analyses was conducted to examine the contribution of participants’ level of socioemotional/instrumental support availability in explaining adjustment and the moderating role of emotional/instrumental support needs. Analyses were run separately for socioemotional and instrumental support availability and support needs in explaining either general, interaction or work adjustment. Summaries of the analyses with emotional support and instrumental support are presented in Tables 3 and 4, respectively. As noted, moderation analyses were performed in line with the recommendations of Holmbeck (1997, 2002). To reduce the effects of multicollinearity, continuous variables were centred (Holmbeck, 1997, 2002). In the present study, 4 blocks of independent variables were entered hierarchically in each linear regression: (1) participants gender and age, (2) time in Belgium, (3) (socioemotional or instrumental) support availability and needs, and (4) the cross-product terms of the respective type of available support and support needs. The variance-inflation factors of all 6 regression analyses were acceptable (range 1.07–2.29), suggesting that there was no problem of multicollinearity. Below, we first report on the analyses with socioemotional support and then report on the analyses with instrumental support.

**Table 3:** Moderation analysis explaining general, interaction and work adjustment by socioemotional support availability and emotional support needs. Standardized betas from the last step in the analyses are displayed.

Criterion variable	Step	Predictor	*β*		R^2^Change		Adj R^2^	

General Adjustment	1	Age	–.04		.05		.02	
		Sex	.11					
	2	Time in Belgium	.36	*	.07	*	.08	*
	3	Socioemotional support availability	.22		.04		.10	
		Emotional support needs	–.14					
	4	Socioemotional support availability × emotional support needs	.14		.02		.11	

Interaction Adjustment	1	Age	–.27	*	.00		–.02	
		Sex	.07					
	2	Time in Belgium	.58	**	.23	*	.20	*
	3	Socioemotional support availability	.25	*	.07	*	.26	*
		Emotional support needs	.05					
	4	Socioemotional support availability × emotional support needs	.02		.00		.25	

Work Adjustment	1	Age	.23		.09	*	.07	*
		Sex	.16					
	2	Time in Belgium	.07		.00		.06	
	3	Socioemotional support availability	.28	*	.07		.10	
		Emotional support needs	–.20					
	4	Socioemotional support availability × emotional support needs	.02		.00		.09	

^(*)^ *p* < .10, * *p* < .05; ** *p* < .001.

**Table 4:** Moderation analysis explaining general, interaction and work adjustment by instrumental support availability and instrumental support needs. Standardized betas from the last step in the analyses are displayed.

Criterion variable	Step	Predictor	*β*		R^2^Change		Adj R^2^	

General Adjustment	1	Age	–.06		.05		.02	
		Sex	.08					
	2	Time in Belgium	.36	*	.07		.08	*
	3	Instrumental support availability	.20		.04		.10	
		Instrumental support needs	–.11					
	4	Instrumental support availability × instrumental support needs	.23	*	.05	*	.14	*

Interaction Adjustment	1	Age	–.34	*	.00		–.02	
		Sex	.09					
	2	Time in Belgium	.62	**	.23	**	.20	
	3	Instrumental support availability	.18		.05		.23	
		Instrumental support needs	.05					
	4	Instrumental support availability × instrumental support needs	.18		.03		.26	

Work Adjustment	1	Age	.22		.09		.07	
		Sex	.17					
	2	Time in Belgium	.08		.00		.06	
	3	Instrumental support availability	.22		.06		.10	
		Instrumental support needs	–.21					
	4	Instrumental support availability × instrumental support needs	.07		.01		.09	

^(*)^ *p* < .10, * *p* < .05; ** *p* < .001.

**Socioemotional support.** Moderation analyses with expatriates’ socioemotional support availability and emotional support needs as independent variables and either general, interaction or work adjustment as dependent variable revealed that, for all three regression analyses, socioemotional support availability had a positive contribution in explaining expatriate adjustment. Analyses indicated that socioemotional support availability was significant in explaining interaction (*β* = .25, *p* < .05) and work adjustment (*β* = .28, *p* < .05) but failed to reach significance for general adjustment (*β* = .22, *p* = .06). Furthermore, there was also a significant positive contribution of time as expatriate in Belgium upon general (*β* = .36, *p* < .05) and interaction adjustment (*β* = .58, *p* < .001), with longer time in Belgium being associated with higher general and interaction adjustment. Age contributed significantly negatively to interaction adjustment (*β* = –.27, *p* < .05). No moderation effect was found for emotional support needs upon the three dimensions of adjustment in understanding the role of socioemotional support availability.

**Figure 1:**
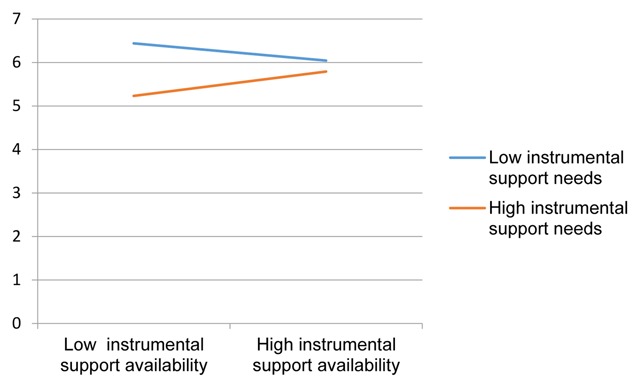
Regression lines for the relationship between the instrumental support availability and general adjustment as moderated by the instrumental support needs. Standardized Beta’s (β) are shown. *β* = –1.14, *p* = .06. *β* = 1.55, *p* < .005.

**Instrumental support.** Similar moderation analyses as reported above but with instrumental support availability and instrumental support needs revealed, similar to the analyses with socioemotional support, a significant positive contribution of time as expatriate in Belgium upon general and interaction adjustment (both *β* ≥ .36, *p* < .05) and a significant negative effect of age upon interaction adjustment (*β* = –.34, *p* < .05). Interestingly, analyses also revealed a significant interaction effect for instrumental support availability × instrumental support needs upon general adjustment (*β* = –.23, *p* < .05). To illustrate the pattern reflected in the statistically significant interaction term, we plotted regression lines for high (+1 SD above the mean) and low (–1 SD below the mean) values of the moderator variable (see e.g., Holmbeck, 1997, 2002) (see Figure 1). Significance tests for both slopes indicated that higher levels of instrumental support availability were associated with higher levels of general adjustment, but only for participants who also reported to have high needs for instrumental support (*β* = 1.55, *p* < .005). The opposite pattern was observed for those who reported low levels of needs for instrumental support; i.e., higher levels of instrumental support availability were associated with worse general adjustment. While this latter pattern failed to reach significance (*β* = –1.14, *p* = .06), the observed interaction shows that, depending upon whether participants reported to have a high need for instrumental support or low need, the impact of instrumental support availability upon general adjustment is opposite to each other. A similar instrumental support availability × instrumental support needs effect was observed for interaction adjustment, but failed to reach significance (*β* = –.18, *p* = .07).

## Discussion

### Summary of Findings

The present study aimed to examine the interrelations between support processes, adjustment, and psychological distress in a sample of help-seeking expatriates living in Brussels. Our results were partially in line with our expectations and can be summarized as follows. First, findings indicated that expatriate adjustment was associated significantly to psychological distress, yet, this was only the case for work adjustment. More specifically, lower levels of expatriates’ work adjustment were associated with higher levels of psychological distress (i.e., depression, anxiety and stress). No significant link to psychological distress was observed for expatriates’ general and interaction adjustment. Second, our analyses exploring the role of social support in expatriates’ adjustment revealed that perceived availability of socioemotional support had a positive association to interaction and work adjustment. Thus, participating expatriates who reported to know more people who could comfort them when feeling homesick or listen to their feelings of loneliness, or share good and bad times with them, felt better adjusted to their work situation and to interacting with Belgian nationals. This was not the case, however, for general adjustment (i.e., life in the new living conditions in general). Third, findings showed that higher levels of perceived instrumental support availability were associated with better general adjustment, but only for expatriates reporting high needs for instrumental support. In other words, the match between the degree of instrumental support expatriates perceived to be available and the amount of instrumental support they tended to seek when dealing with their situation appeared more important for their adjustment to everyday living conditions in the new country (i.e., general adjustment) than the mere availability of instrumental support.

Our findings indicating a negative association between cross-cultural work adjustment and psychological distress, but not between general/interaction adjustment and psychological distress, cannot be directly compared to existing findings as no research on expatriates thus far has been conducted with help-seeking samples to examine this association. However, our findings might be interpreted in light of previous studies showing that work and career issues are central in expatriates’ lives. For example, work/career opportunities are one of the most common reasons why expatriates decide on expatriate experience (Dickmann et al., 2008; Selmer & Lauring, 2011) and expatriates often have high status jobs (Silbiger & Pines, 2014). Furthermore, high perceived work importance was found to aggravate the impact of stress on burn out (Silbiger & Pines, 2014). Accordingly, this may explain why psychological distress was associated with work adjustment but not with general and interaction adjustment.

It has been argued, however, that the process of work adjustment might be more straightforward or even easier than in other domains of adjustment (Hippler et al., 2015). But what happens when this is not the case? Our study findings show that this doesn’t hold for the expatriates who already experience psychological distress. Previous findings have shown that poor work adjustment can lead to personal costs (Firth et al., 2014). As the average duration of stay in the host country in our study was 8.7 years it may be assumed that by such length of stay expatriates should already reach a level of successful adjustment. Indeed, according to recent conceptualizations, the adjustment for company sponsored expatriates should reach its peak after 2–5 years (Hippler et al., 2015) or slightly later (Bhaskar-Shrinivas et al., 2005). However, the adjustment processes may run differently and at different pace on respective adjustment domains. Our results suggest that this may indeed be the case as work adjustment for our sample was associated with higher psychological distress whereas such an association was not found in general and interaction domains of adjustment and psychological distress. This means that longer-term expatriates – which was the case in our sample – may experience more fluctuations within their adjustment process, and perhaps reach more ‘downs’ and ‘highs’ points of their adjustment curve, the latter not completely corresponding to the adjustment curves that the literature has presented so far (Bhaskar-Shrinivas et al., 2005; Black & Mendenhall, 1990).

Our study also documents a positive association between perceived availability of socioemotional support on the one hand and interaction and work adjustment on the other hand. This points to the importance for expatriates to feel comforted and helped with their specific emotional needs in order to open up and relate to locals (cf. interaction adjustment) or even co-workers (cf. work adjustment), who in turn can then become important sources of support (Aycan, 1997; Podsiadlowski et al., 2013). These findings corroborate previous studies emphasizing that perceived availability of quality rather than quantity of social interactions and support is crucial for expatriates’ mental health (McGinley, 2008). For help-seeking expatriates, psychotherapy offers the opportunity to discuss specific psychological challenges such as identity issues, questions of belonging, homesickness, rootlessness, repeated goodbyes, and unresolved grief (Bushong, 2013). By receiving socioemotional support from their therapists, and also colleagues, friends and family, they could thus be able to assess their life expectations and to adjust their beliefs in order to see their current expatriate situation in a more positive way. The perceived availability of socioemotional support may make them feel more confident to reach out and to learn new people and connect and interact with them in their immediate environment (Szabo, Ward & Jose, 2016).

It is important, however, that clinicians working with expatriates have additional training in understanding the nature of the complexity of expatriate lifestyle and the specific challenges that are part of expatriate life (i.e., uprooting, constant changes, and repeated goodbyes). Having their own expatriate experience seems to be an asset for psychotherapists (Bushong, 2013). This supports the finding of our study, that receiving adequate socioemotional support is associated with their better (interaction and work) adjustment which in turn can help them to accept the hardships of their work situation and increase work adjustment. That, as a consequence, would ease their psychological distress, as supported in our first hypothesis.

Our moderation analysis indicated that perceived availability of instrumental support fosters general adjustment (e.g., food, shopping, health care, transport, etc.), particularly for those expatriates who are in need for this kind of support. The role of instrumental support for expatriate adjustment has previously been recognized in the research (McGinley, 2008; Selmer, 1999). It is also in accordance with contemporary social support research and intervention literature (Melrose et al., 2015; Rafaeli & Gleason, 2009) documenting the importance of support provision matching the recipient’s need for a specific type of support. Providing sufficient and matching instrumental social support to expatriates’ needs seems to be key. What does that truly mean for the expatriates already experiencing psychological distress? A foreign environment may feel overwhelming to expatriates due to the novelty and host country languages and culture, even when instrumental support is available. As exciting as it can be to live in a foreign culture, one has to have a “compass” as to how to use all the potentially available instrumental support. Namely, one of the reasons for such a paradox may be that expatriates don’t particularly know how to actually use the available support (Rosenbusch, Cerny II, & Earnest, 2015). On the other hand, expatriates with previous international work experience or longer-term expatriates- which was the case in our study- may be in a better position as they may be more skilled in recognizing their own needs for the particular type of support. This is also due to their lifestyle full of changes and adjustments (Bushong, 2013), as they may have learned what works well for them and as a result they are more conscious of their particular needs. The optimal matching theory therefore provides a plausible explanation for the moderating role of social support needs in understanding the impact of perceived support availability (Cutrona & Russell, 1990). Specifically, mismatched support could compromise feelings of autonomy, competence and feelings of independence and self-sufficiency (Rafaeli & Gleason, 2009), all being very important in supporting expatriate complexity and the associated psychological challenges that it entails (Bushong, 2013; Filipič Sterle et al., 2018b). In other words, as the results of our study suggest, being more aware of expatriates’ own needs and the kind of help they would need, such as asking for advice and support from other people (cf. instrumental support), may help them to make an effort to meet them. While socioemotional support availability was found to have a positive association with expatriate work and interaction adjustment, no moderation effect was found for emotional support needs. Although it is unclear why this is the case, one tentative explanation is that emotional support needs (i.e., the need for a human contact, comfort, identity issues, homesickness, uprootlessness, etc. (Bushong, 2013) were already addressed during psychotherapy. However, further research is needed to replicate the present findings and/or explore alternative explanations.

Of further interest, our study revealed that the duration of stay in Belgium and gender impacted some of the variables included in the present study. Specifically, expatriates who have lived in Belgium for a longer period, were better adjusted to the life in Belgium in general and had better interaction with Belgian locals. This finding supports previous findings that difficulties in socio-cultural adjustment generally decrease over time (Ward, Okura, Kennedy & Kojima, 1998). Further, age contributed significantly negatively to the interaction adjustment suggesting that younger expatriates showed better interaction adjustment with locals than older expatriates. Interestingly, apart from emotional support needs being higher in women, no additional gender differences were found for the remainder of the support variables. Despite the growing body of evidence documenting that women tend to be better adapted than men overall, and significantly so in the interaction adjustment (Haslberger, 2010), our study has not supported these findings. Previous studies have also argued that there might be some differences in how men and women experience expatriate experiences. For instance, in cultures with sex-role expectations that are very different from the home culture, women may find opportunities for social interaction to be limited (Fontaine, 1986). The latter might have been less a problem in the current sample as the majority of study participants were coming from other European countries and living in Brussels, which is a very international environment. The finding that women showed a greater need for emotional support than men is in line with the consensus in the general and multicultural literature, documenting that seeking emotional support is a particularly important coping strategy for women when dealing with problems (Jackson, 2006; Madhyastha, Latha & Kamath, 2014). Furthermore, in most cultural contexts women are taught to rely on other people, particularly for emotional support (Jackson, 2006).

### Strengths and Limitations

The present study has a number of strengths. First, this is the first study that examined expatriate adjustment, psychological distress and perceived social support availability and needs within a sample of help-seeking expatriates who showed severe levels of psychological distress and who were all undergoing psychotherapy treatment at the time of the study. As such, the present study sheds light on the processes that make some expatriates particularly vulnerable for psychological problems. Second, our study used different theoretical models from acculturation and expatriate adjustment literature, linking it to coping and stress models and specifically, the clinical interventions literature for the globally mobile. Third, by using a multi-dimensional conceptualization and measurement of our study variables, more fine-grained conclusions can be drawn about which specific type of support (i.e., socioemotional vs. instrumental support) is most helpful in fostering adjustment within particular life domains (e.g., general life conditions, interaction with locals, and work situation). Another strength of the current study relates to the cultural diversity of the sample (i.e., participants were coming from 26 different countries of origin), enhancing the generalizability of our findings across cultures.

In addition to the various strengths of the current study, some limitations should be noted, each of which points to directions for future research. First, because of the lack of previous research using samples of help-seeking expatriates, the current findings cannot be fully compared to existing findings derived from other samples of expatriates. Second, our sample was rather small and mainly consisted of women with the majority of participants having a high educational status (i.e., university or academic degree) (*N* = 76). Replication of these findings with larger and more heterogeneous samples will be important. Employing a larger sample would also allow to examine differences across cultures. On the other hand, given the heterogeneity of our sample with a high variation of age of expatriates (i.e., ranging from 19 to 70 years) and a broader definition of expatriates (i.e., also including trailing partners), findings should be interpreted with caution. More research is needed to see whether findings hold for different ‘subgroups’ of expatriates and more homogeneous samples of expatriates, or whether there are substantial differences. Third, while it is likely that a certain number of participants who moved to Belgium years ago as trailing partners have found themselves a job, this was not explicitly checked in our study. Forth, it should be noted that the temporal order of the processes under investigation cannot be tested. The possibility exists that psychological problems in expatriates lead to lower levels of adjustment, rather than the other way around. Caution should therefore be exercised in inferring causality from our results. Given the cross-sectional design of our study, changes of adjustment levels on different domains, at different times could not be tested therefore we are limited to the conclusion that work and psychological distress are significantly linked, even after the average stay of more than eight years. Fifth, the level of adjustment within our sample of help-seeking expatriates was moderate (i.e., their responses ranged between “adjusted a little” and “generally adjusted” for all three levels of adjustment). Further research will be needed using samples that are less well-adjusted. As a fruitful avenue for future research it is suggested to include comparative samples with expatriates who are not seeking psychological help to examine the differences in coping strategies. Finally, it will be important to supplement the current survey findings with research using alternative methods of assessing support, adjustment and distress. In particular, qualitative research methods may provide a more in-depth understanding of expatriates’ experiences (e.g., specific stressors, specific sources of support) (Filipič Sterle et al., 2018b).

## Conclusion

The current study is the first study using a sample of help-seeking expatriates to explore the association between perceived social support, psychological distress, and adjustment. Findings suggest that expatriates’ successful work adjustment is important as it is linked to psychological distress. Available emotional support from different sources fosters expatriates’ interaction and work adjustment. And finally, expatriates are better adjusted in everyday living conditions when provided with instrumental support matching their needs. It is therefore of key importance that adequate professional support is offered to expatriates who already show increased level of psychological distress, such as stress, anxiety, depression that can lead to potential burn-out. Access to psychological counselling can be a good way to alleviate distress and put emotional support into function. Additionally, making new connections and integration of expatriates in a new environment to use other kinds of support, particularly to be able to ask other people for help, should receive more focus.
